# Thrust Improvement of a Biomimetic Robotic Fish by Using a Deformable Caudal Fin

**DOI:** 10.3390/biomimetics7030113

**Published:** 2022-08-14

**Authors:** Hua Shao, Bingbing Dong, Changzhen Zheng, Te Li, Qiyang Zuo, Yaohui Xu, Haitao Fang, Kai He, Fengran Xie

**Affiliations:** 1 Key Laboratory of Metallurgical Equipment and Control Technology, Ministry of Education, Wuhan University of Science and Technology, Wuhan 430081, China; 2 Hubei Key Laboratory of Mechanical Transmission and Manufacturing Engineering, Wuhan University of Science and Technology, Wuhan 430081, China; 3 Precision Manufacturing Institute, Wuhan University of Science and Technology, Wuhan 430081, China; 4 Shenzhen Institutes of Advanced Technology, Chinese Academy of Sciences, Shenzhen 518055, China; 5 College of Mechanical Engineering, Guangxi University, Nanning 530004, China; 6 Shenzhen Key Laboratory of Precision Engineering, Shenzhen 518055, China; 7 School of Computer Science and Control Engineering, University of Chinese Academy of Sciences, Beijing 100049, China; 8 School of Artificial Intelligence, Shenzhen Polytechnic, Shenzhen 518055, China

**Keywords:** biomimetic robotic fish, deformable caudal fin, thrust generation

## Abstract

In nature, live fish has various deformable fins which are capable to promote the swimming speed, efficiency, stability, and thrust generation. However, this feature is rarely possessed by current man-made biomimetic robotic fishes. In this paper, a novel deformable caudal fin platform is proposed to improve thrust generation of biomimetic robotic fish. First, the design of the deformable caudal fin is given, which includes a servo motor, a gear-based transmission mechanism, fin bones, and silica membrane. Second, an improved Central Pattern Generator (CPG) model was developed to coordinately control the flapping of the tail and the deformation of the caudal fin. More specifically, three deformation patterns, i.e., conventional nondeformable mode, sinusoidal-based mode, instant mode, of the caudal fin are investigated. Third, extensive experiments are conducted to explore the effects of deformation of the caudal fin on the thrust generation of the biomimetic robotic fish. It was found that the instant mode of the caudal fin has the largest thrust, which sees a 27.5% improvement compared to the conventional nondeformable mode, followed by the sinusoidal-based mode, which also sees an 18.2% improvement. This work provides a novel way to design and control the deformation of the caudal fin, which sheds light on the development of high-performance biomimetic robotic fish.

## 1. Introduction

Most fish generate thrust by passing a traveling wave of increasing amplitudes from head to tail. This kind of fish is known as the Body and/or Caudal Fin (BCF) swimmer [1,2]. For a BCF swimmer, the posterior part of the fish body is much more important than the anterior part for swimming locomotion since it is where most of the thrust comes from. As a result, a careful design of the posterior part, especially the caudal fin, is essential for developing a high-performance biomimetic robotic fish, which is useful in some applications such as narrow space navigation, low-noise surveillance, and environment monitoring.

At present, studies about the caudal fin’s flapping pattern, stiffness, and shape are attracting researchers’ attention. Isolating effects of such parameters is difficult in live fish. As a result, robotic models are developed to provide an alternative way. For example, Xie et al. examined how tail’s flapping patterns (with different amplitudes, frequencies, asymmetry, and shape parameters) affected fish swimming performances, such as the cruising speed, the recoil, the thrust generation, and swimming efficiency [3]. Notably, they found that the sinusoidal flapping pattern of the caudal fin, which was also adopted in Lighthill’s Elongated Body Theory (EBT) [4,5], had a good balance among the thrust generation, the recoil, and the swimming speed, which resulted in a high swimming efficiency. The stiffness also has a significant effect on swimming performances. Wolf et al. developed a pneumatically actuated fish-like model to study the role of stiffness on locomotor thrust generation. They showed that both the thrust and lateral force rose with the increase in frequency for the stiffer model. The stiffer the tail, the more impact the increasing frequency had on thrust generation. Moreover, flexural stiffness falls along fish’s anterior-posterior axis in nature [6]. In order to examine the role of non-uniform bending stiffness during fish swimming, Lucas et al. fabricated foil models with discrete regions of high (5.5 × 10^−5^ Nm^2^) and low (1.9 × 10^−5^ Nm^2^) flexural stiffness of biologically relevant magnitudes. In comparison to the uniform distributions of stiffness, the combination of non-uniform stiffness distributions and 0° angle of attack pitching program better mimicked the kinematics of live fish swimming; thus, it also had better swimming performances in terms of speed, efficiency, and thrust generation [7]. Matta et al. compared three shapes of the caudal fins, i.e., rectangular, elliptical, and swept (lunar). It was found that the lunar caudal fin, most similar to a fusiform swimmer, had the largest thrust, followed by the elliptical fin. The rectangular caudal fin generally generated the least thrust [8,9,10]. Similar conclusions can also be found in [11,12,13,14,15].

The above studies shed light on the role of caudal fin’s flapping pattern, stiffness, and shape on the swimming performances. However, the stiffness and shape of the caudal fin usually cannot be changed when the robotic fish is freely swimming. In contrast, live fish is capable of modulating the stiffness or shape during swimming in real-time to adapt to surrounding aquatic environment [16,17,18,19]. Accordingly, novel mechanisms to adjust stiffness are developed for biomimetic robotic fishes in these years. Chen et al. designed and fabricated two tensegrity robotic fishes, one of which was based on tensegrity joints by means of tension elements [20], and the other one of which was based on antagonistic stiffness that resulted from the prestress of tension structures in a kinematically singular configuration [21]. Park et al. proposed a novel variable-stiffness flapping (VaSF) mechanism for a biomimetic robotic dolphin. This mechanism was made up of compliant and rigid segments alternately in series, and two tendons run through it to adjust stiffness [22]. In order to decouple the adjustable stiffness from the inherent stiffness, Li et al. proposed a stiffness decoupled mechanism based on the Mechanically Adjustable Compliance and Controllable Equilibrium Position Actuator (MACCEPA), and used it to construct a soft biomimetic robotic fish with large stiffness variation [23]. Zhong et al. developed a variable-stiffness experimental platform and explained how and why tuning stiffness affected performances. Notably, they found that the stiffness should be scaled with swimming speed squared to maximize the swimming speed, which provided a simple stiffness tuning strategy for biomimetic robotic fish [24]. In comparison to stiffness-variation mechanisms, studies about mechanisms to online modulate the shape of a caudal fin are far less. A caudal fin with a hole and a moving cover on it was developed. By actuating the moving cover, the area of the caudal fin could be adjusted [25,26]. Tangorra and Lauder [27,28,29] developed several robotic fins, including the caudal fin, to study how fish produced and controlled forces, finding that subtle changes to the kinematics and mechanical properties of fin rays can significantly impact the magnitude, direction, and time course of the 3D forces used for propulsion and maneuvers. More specifically, the deformable caudal fin was made up by six individually moveable fin rays, and five kinematic patterns were examined, i.e., flat movement of the entire fin, cupping of the fin, W-shaped fin motion, fin undulation and rolling movements. Notably, the cupping motion produced the largest thrust. However, area change of the caudal fin is not investigated in their works. Yang et al. used a crank slider mechanism to design a caudal fin capable of deform slowly among circular, trapezoid, and lunar shapes [30]. Mechanisms to modulate the area can also be found in the design of other biomimetic robots. Tang [31] and Pandy [32] used the dielectric elastomer actuator and a slider mechanism to change the area of webbed feet of a biomimetic robotic frog. However, among these studies, one common limitation is that how to coordinately control the deformation of caudal fin and the flapping of tail propeller within one flapping cycle has not been studied. Moreover, most of the deformation of the caudal fin is not biomimetic. In nature, there rarely exists fish with a hole on the caudal fin, or with a trapezoid/square-shaped caudal fin. 

In this paper, a novel deformable caudal fin platform is proposed to improve the thrust generation of biomimetic robotic fish. The contributions of this paper are twofold. On one hand, the design and control strategy of the deformable caudal fin are firstly proposed. The design offers a quick deformation ability for the caudal fin, and an improved Central Pattern Generator (CPG) model provides coordinate control between the flapping of the tail and the deformation of the caudal fin. More specifically, three deformation patterns, i.e., conventional nondeformable mode, sinusoidal-based mode, instant mode, of the caudal fin are investigated. On the other hand, by using this novel deformable caudal fin, the thrust sees a 27.5% improvement compared with conventional nondeformable caudal fin with proper deformation control strategy. Since measuring the real-time thrust when the robotic fish is freely swimming is very difficult, in this paper, we use ‘stationary thrust’ to estimate the ‘real thrust’. The stationary thrust is obtained when the robotic fish is fixed to a load cell.

The rest of this paper is organized as follows. Section 2 introduces materials and methods, including the design and CPG control strategy. Section 3 gives experiments of three deformation patterns, i.e., conventional nondeformable mode, sinusoidal-based mode, and instant mode. Section 4 provides a detailed discussion of the experimental results. Finally, Section 5 concludes this article and gives an outlook on the future research direction.

## 2. Materials and Methods

### 2.1. The Design of the Deformable Caudal Fin

**Figure 1:**
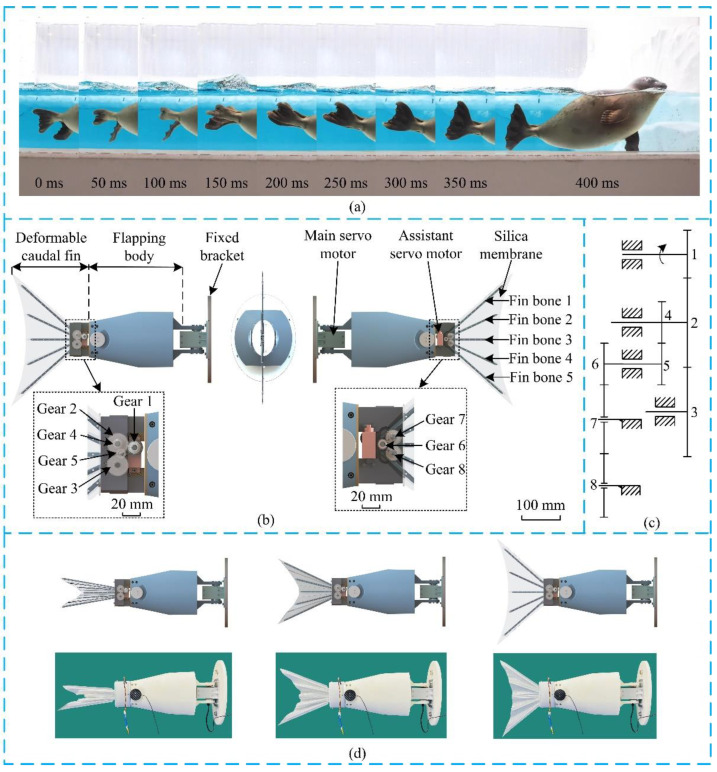
The design of the deformable caudal fin: (**a**) Snapshots of a swimming seal’s webbed feet [35]. (**b**) CAD model of the deformable caudal fin. (**c**) Gear-based transmission mechanism. (**d**) The prototype of the deformable caudal fin platform and states of a folded caudal fin becoming unfolded.

In nature, both of the fish and the seal are excellent swimmers. In this paper, on one hand, the basic design of the robotic caudal fin is based on fish, such as the lunar shape, the number of fins, etc. On the other hand, inspired by seal, we incorporate the fish’s caudal fin with deformation ability. According to [10,33,34], during one flapping cycle, the caudal fin of robotic fish generates both thrust and drag. In order to increase the mean driving force, the caudal fin is designed to rise its area during the time slice that generates thrust, and reduce the area during the time slice that produces drag. As shown in Figure 1a [35], this area modulation feature is exactly possessed by the seal, instead of other types of fishes.

Inspired by this, the deformable caudal fin for a biomimetic robotic fish is designed, which is as shown in Figure 1b. The platform mainly contains three parts, i.e., a fixed bracket as the base, a flapping body, and the deformable caudal fin. One main servo motor (DYNAMIXEL XW540-T140-R) is used to directly drive the flapping body. When the main servo motor rotates back and forth, the flapping body generates periodical movement. In addition, one assistant servo motor (Hitec HS-5086WP) is used to power the deformation of the caudal fin. The deformable caudal fin includes a gear-based transmission mechanism, five fin bones, and a silica membrane. Specifically, Fin bone 1, Fin bone 2, Fin bone 4, and Fin bone 5 are mounted on Gear 2, Gear 7, Gear 8, and Gear 3, respectively. Fin bone 3 is fixed. The gear-based transmission mechanism is detailed in Figure 1c, where the gear ratios *z*_1_:*z*_2_:*z*_3_:*z*_4_:*z*_5_:*z*_6_:*z*_7_:*z*_8_ = 30:50:50:24:24:24:36:36. It is seen that the rotational speed of Fin bone 1 ($\omega_{F1}$) and the rotational speed of Fin bone 5 ($\omega_{F5}$) are identical, with the speed ratio to the assistant motor of 3:5. Similarly, the rotational speed of Fin bone 2 ($\omega_{F2}$) and the rotational speed of Fin bone 4 ($\omega_{F4}$) are the same, with the speed ratio to Fin bone 1 or 5 of 2:3. Overall, the fin bones in the outer side of the caudal fin move faster than the fin bones in the inner side. The speed ratios of the five fin bones are $\omega_{F1}$:$\omega_{F2}$:$\omega_{F3}$:$\omega_{F4}$:$\omega_{F5}$ = 3:2:0:2:3. One characteristic of this design is that the caudal fin is capable to mimic its counterpart in nature, maintaining lunar shape during the whole deformation process, and strong enough to interact with surrounding aquatic environment. Figure 1d shows the prototype and three states of a folded caudal fin becoming unfolded.

### 2.2. CPG Control and Deformation Patterns

Central Pattern Generator (CPG) is one of the most widely adopted control methods in biomimetic robotic fishes. It coordinates movements among different joints, and facilitates transitions among different states. In this paper, an improved CPG model is developed from Ijspeert’s salamander robot [36,37]. Similar work can also be found in [3]. It coordinately controls flapping of the fish body and deformation of the caudal fin, which differs from most of the other CPG control among various joints. 

The improved CPG model starts as follows:

(1) $${\ddot{c}}_{i}=k_{c}(0.25k_{c}(C_{i}-c_{i})-{\dot{c}}_{i})$$

(2) $${\ddot{a}}_{i}=k_{a}(0.25k_{a}(A_{i}-a_{i})-{\dot{a}}_{i})$$

(3) $${\ddot{g}}_{i}=k_{g}(0.25k_{g}(G_{i}-g_{i})-{\dot{g}}_{i})$$

(4) $${\dot{\phi }}_{i}=2\pi f_{i}$$

(5) $$\alpha_{i}=c_{i}+a_{i}\cos(\phi_{i}-g_{i}\cdot 2\pi )$$

where i denotes the *i*th oscillator, i = 1, 2 in this paper, the 1st oscillator (CPG 1) corresponds to the fish body and the 2nd oscillator (CPG 2) corresponds to the caudal fin. $c_{i}$ is the offset state, $C_{i}$ is the offset command, $k_{c}$ is a positive constant representing how fast $c_{i}$ converges to $C_{i}$. $a_{i}$ is the amplitude state, $A_{i}$ is the amplitude command, $k_{a}$ is a positive constant representing how fast $a_{i}$ converges to $A_{i}$. $g_{i}$ is the phase difference state, $G_{i}$ is the phase difference command, $k_{g}$ is a positive constant representing how fast $g_{i}$ converges to $G_{i}$. $\phi_{i}$ represents the phase state, $f_{i}$ is the control command of frequency. $\alpha_{i}$ is the output of the oscillator.

For CPG 1, since the fish body flaps symmetrically, the offset command $C_{1}$ is set to 0. Meanwhiles, the phase difference command $G_{1}$ is also set to 0. Thus, the output of CPG 1, $\mu_{1}$, is:

(6) $$\mu_{1}=\alpha_{1}=a_{1}\cos(\phi_{1})$$

**Figure 2:**
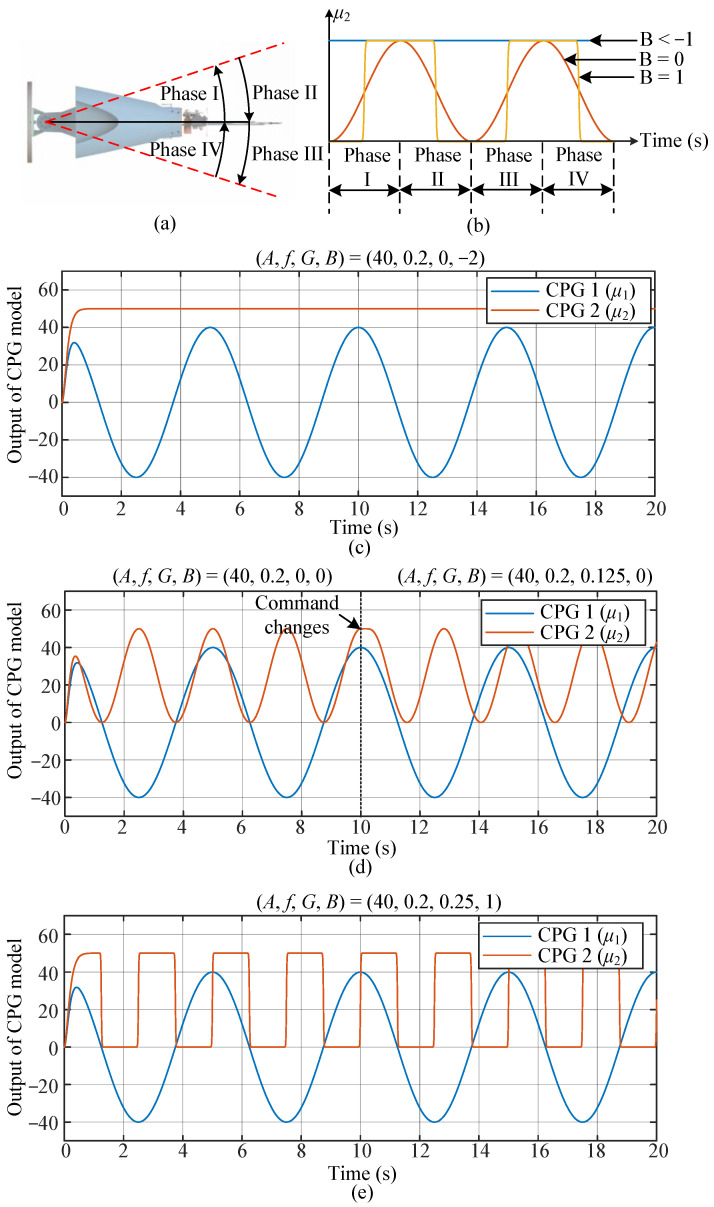
Deformation patterns: (**a**) Four phases of a flapping cycle. (**b**) Output signal of CPG 2. (**c**) Conventional nondeformable mode. High-level control command (*M*, *f*, *G*, *B*) = (40, 0.2, 0, −2). (**d**) Sinusoidal-based mode. High-level control command (*M*, *f*, *G*, *B*) = (40, 0.2, 0, 0) → (40, 0.2, 0.125, 0) at *t* = 10 s. (**e**) Instant mode. High-level control command (*M*, *f*, *G*, *B*) = (40, 0.2, 0.25, 25).

For CPG 2, one flapping cycle can be divided into four phases, i.e., Phase I to Phase IV, which is as shown in Figure 2a. Normally, Phase I and Phase III are called the beat phase, and Phase II and Phase IV are called the restore phase. Deformation of the caudal fin in Phase I is identical to that of Phase III, and deformation of the caudal fin in Phase II is the same with that of Phase IV. In other words, deformation of the caudal fin is symmetrical about main axis of the fish body. Thus, the frequency control command $f_{2}$ is set to $2f_{1}$. Moreover, the amplitude control command and offset control command of CPG 2 are set to be identical, i.e., *A*_2_ = *C*_2_.

To realize a more diversified deformation pattern of the caudal fin, the output of oscillator 2, $\alpha_{2}$, is further processed by following equations.

(7) $$\ddot{b}=k_{b}(0.25k_{b}(B-b)-\dot{b})$$

(8) $$\mu_{2}=\left\{ \begin{array}{ll} \frac{a_{2}\cdot \tanh(\frac{b}{a_{2}}(\alpha_{2}-c_{2}))}{\tanh(b)}+c_{2},\; & b>0\\ \alpha_{2}\;,\; & b=0\\ \frac{a_{2}\sin^{-1}(-\frac{b}{a_{2}}\cdot (\alpha_{2}-c_{2}))}{\sin^{-1}(-b)}+c_{2}, & -1\leq b<0\\ 2a_{2}, & b<-1 \end{array}\right.$$

where $\mu_{2}$ is the output of the CPG 2, which is used to drive the deformation of the caudal fin. b is the shape state, B is the shape control command, $k_{b}$ is a positive constant representing how fast b converges to B. As shown in Figure 2b, when *B* = −2, *B* = 0, *B* = 25, the shape of the deformation pattern changes from a straight line to sinusoidal and square, respectively.

Overall, the high-level control command of the improved CPG model is set to be (*A*, *f*, *G*, *B*), where *A* is the amplitude control command determining flapping amplitude of the fish body, *f* is the frequency control command determining flapping frequency of the fish body, *G* is the phase difference control command determining the phase difference between flapping of fish body and deformation of the caudal fin (which is identical to $G_{2}$), *B* is the shape control command. Please note that the caudal fin deforms between its limit positions, as a result, the amplitude and offset control commands for CPG 2, i.e., *A*_2_ and *C*_2_, are not included in the high-level control command.

Based on the improved CPG model above, we formulate three deformation patterns of the caudal fin, which are shown in Table 1.

When *B* = 0, the deformation pattern is defined as sinusoidal-based mode, where the output of CPG 2 ($\mu_{2}$) is a sinusoidal signal (as shown in Figure 2d). The phase difference between the flapping of the fish body and the deformation of the caudal fin is determined by *G*. For example, in Figure 2d, the phase difference control command (*G*) is changed from 0 to 0.125 at *t* = 10 s, it is found that the phase difference between CPG 1 (the blue line) and CPG 2 (the red line) is changed, correspondingly.

When *B* = 25, the deformation pattern is defined as instant mode, where the output of CPG 2 ($\mu_{2}$) is a square signal (as shown in Figure 2e). In this paper, *G* is set to 0.25, as a result, the caudal fin switches to folded state at the beginning of the beat phase (Phase Ⅰ and Phase Ⅲ), and then switches to unfolded state at the beginning of the restore phase (Phase Ⅱ and Phase Ⅳ).

## 3. Experiments

**Figure 3:**
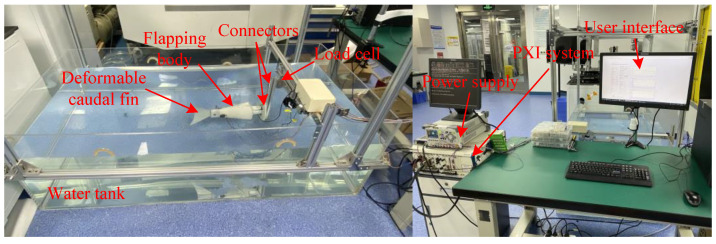
Experimental Platform.

An experimental platform is developed to test thrust generation of the biomimetic deformable caudal fin, which is as shown in Figure 3. The deformable caudal fin is fixed on a load cell (model: DYLY-102) through connectors. The load cell obtains thrust and sends the data back to a data acquisition board (model: PXI-6289), which is installed on a PXI system. The PXI system is also equipped with a controller (model: PXI-8106) for real-time control, generating CPG signals to drive two servo motors. Moreover, there is one programmable power supply (model: DP832A). The water tank is 2 m in length, 1.0 m in width, and 0.6 m in height. The user interface (UI) running the computer is programed by LabVIEW.

### 3.1. Conventional Nondeformable Mode

In the conventional nondeformable mode, the caudal fin stays unfolded when the fish body flaps, which is the same as most other caudal fins. The high-level control commands are as shown in Table 2. More specifically, the shape control command (*B*) is −2, and the phase difference control command (*G*) is 0. There are totally four amplitudes (35°, 40°, 45°, 50°) and three frequencies (0.20 Hz, 0.25 Hz, 0.30 Hz) to be tested.

**Figure 4:**
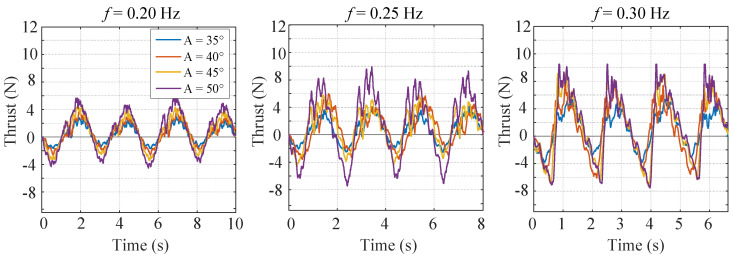
Experimental results of conventional nondeformable mode.

The experimental results are as shown in Figure 4. One general trend is that both the positive thrust and the negative thrust rise with the increase of flapping amplitude and flapping frequency. As a result, the mean thrust does not increase so much since the growing negative thrust counteracts the growing positive thrust. It is also found that the frequency of the force is twice the flapping frequency. This is because the fish body flaps symmetrically about the main axis. Moreover, due to the water fluctuation, high frequency components grow with the increase of the flapping frequency and the flapping amplitude.

### 3.2. Sinusoidal-Based Mode

In the sinusoidal-based mode, the caudal fin conducts a sinusoidal deformation pattern, similar with the flapping of the fish body. The high-level control commands are as shown in Table 3. More specifically, the shape control command (*B*) is 0, and the phase difference control command (*G*) varies from 0 to 7/8 with an interval of 1/8. Four amplitudes (35°, 40°, 45°, 50°) and three frequencies (0.20 Hz, 0.25 Hz, 0.30 Hz) are examined. Thus, there are totally 96 (=3 × 4 × 8) sets of experiments. Each set of experiments is conducted five times.

**Figure 5:**
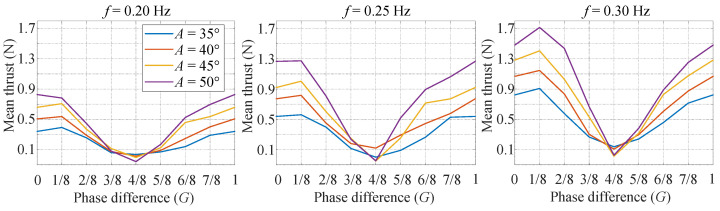
Experimental results of sinusoidal-based mode.

The mean thrust is shown in Figure 5, which sees a ‘V’ shape. One interesting finding is that the phase difference *G* = 4/8 has the smallest mean thrust for all the tested frequencies and amplitudes. Some tests even have a negative value. The reason is that the caudal fin deforms with small area during the time slice producing positive thrust, while deforms with large area during the time slice generating negative thrust. In contrast, it is found that the phase difference near *G* = 1/8 has the largest mean thrust.

### 3.3. Instant Mode

In the instant mode, the caudal fin switches to folded state at the beginning of the beat phase (Phase Ⅰ and Phase Ⅲ), and then switches to unfolded state at the beginning of the restore phase (Phase Ⅱ and Phase Ⅳ). The high-level control commands are as shown in Table 4. More specifically, the shape control command (*B*) is 25, and the phase difference control command (*G*) is 0.25. There are also four amplitudes (35°, 40°, 45°, 50°) and three frequencies (0.20 Hz, 0.25 Hz, 0.30 Hz) to be tested.

**Figure 6:**
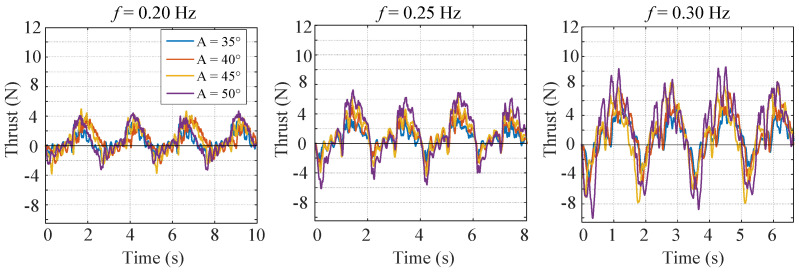
Experimental results of instant mode.

The experimental results are as shown in Figure 6. It is found that the thrust rises with the increase of flapping amplitude and flapping frequency. Moreover, the positive thrust grows more rapidly than the negative thrust, and the time slice for the positive thrust also becomes longer than the time slice for the negative thrust. As a result, the mean thrust rises significantly.

## 4. Discussion

**Figure 7:**
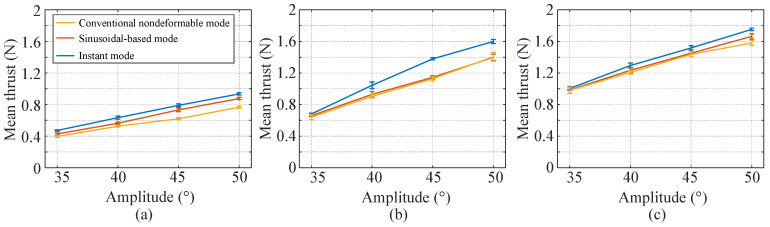
Comparison of mean thrust of the three deformation patterns: (**a**) Mean thrust at *f* = 0.20 Hz. (**b**) Mean thrust at *f* = 0.25 Hz. (**c**) Mean thrust at *f* = 0.30 Hz.

This paper presents a novel deformable caudal fin platform for a biomimetic robotic fish. An improved CPG model is proposed, and three deformation patterns, i.e., conventional nondeformable mode, sinusoidal-based mode, instant mode, are formulated. Comparisons of the mean thrust of the three deformation patterns are made, which is as shown in Figure 7. Each point is the mean thrust of five tests, and each test contains four periods. Please note that the largest mean thrust of sinusoidal-based mode is adopted in the comparison, which means the phase difference is 1/8. From Figure 7, two conclusions can be drawn. (1) The instant mode has the largest mean thrust, followed by the sinusoidal-based mode. The conventional nondeformable mode has the smallest mean thrust. Actually, when the flapping amplitude is small (*A* = 35°), thrust of the three deformation patterns is close. However, thrust of the instant mode augments more rapidly than the other two modes. (2) For all the three deformation patterns, the mean thrust grows with the increase of the flapping amplitude and the flapping frequency.

Table 5 and Table 6 shows the mean thrust of the sinusoidal-based mode and instant mode in comparison to the conventional nondeformable mode, which is most widely employed in current biomimetic robotic fish. It is found that both the sinusoidal-based and the instant mode have a significant improvement. Notably, when the flapping frequency is 0.2 Hz and the flapping amplitude is 45°, the sinusoidal-based mode sees a 18.2% improvement and the instant mode sees a 27.5% improvement, which is a big promotion to the thrust generation of a biomimetic robotic fish.

**Figure 8:**
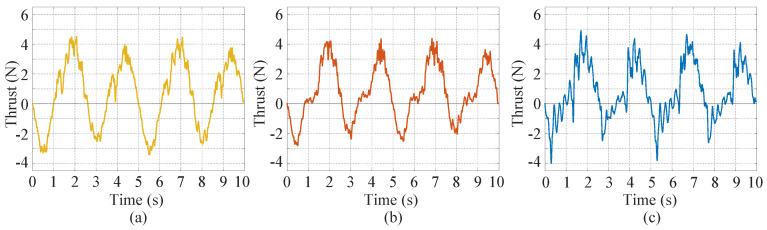
Instantaneous thrust of the three deformation patterns when *A* = 45° and *f* = 0.20 Hz. (**a**) Conventional nondeformable mode. (**b**) Sinusoidal-based mode. (**c**) Instant mode.

A further analysis is given to explain why these three deformation patterns have different thrust generation. Figure 8 shows the instantaneous thrust at the frequency of 0.2 Hz and amplitude of 45°. It is found that even though the peak-to-peak amplitudes of these deformation patterns are close, the positive thrust and negative thrust generated in one flapping cycle are significantly different. The conventional nondeformable mode generates the most negative thrust in one cycle, followed by the sinusoidal-based mode. The instant mode has the least negative thrust. As a result, the instant mode has the largest mean thrust and the conventional nondeformable mode has the least mean thrust. Moreover, it is also found that the instant mode fluctuates more intensively than the other two. The reason may be that this mode contains sudden deformation of the caudal fin, and only the deformation speed (the speed of the assistant servo motor) of the instant mode is discontinuous. Please note that a careful choice of the control parameters is needed to make the deformable caudal fin have better performances. A counter example is that for the sinusoidal-based mode, the caudal fin may generate negative thrust when the phase difference is 4/8.

## 5. Conclusions and Future Work

In this paper, a novel deformable caudal fin platform is presented to improve thrust generation of biomimetic robotic fish. The design and control are detailed. An improved CPG model is developed and three deformation patterns, i.e., conventional nondeformable mode, sinusoidal-based mode, instant mode, are formulated to verify the performance of it. Extensive experiments are carried out. More specifically, diversified combinations of the flapping frequencies (0.20 Hz, 0.25 Hz, and 0.3 Hz), the flapping amplitudes (35°, 40°, 45°, 50°) and the phase differences (0, 1/8, 2/8, 3/8, 4/8, 5/8, 6/8, 7/8) are examined. Overall, it is found that the instant mode of the caudal fin is proven to have the largest thrust, which sees a 27.5% improvement compared to the conventional nondeformable mode, followed by the sinusoidal-based mode, which also sees an 18.2% improvement. In addition, it is also seen that even though the peak-to-peak amplitudes of these deformation patterns are close when the same flapping frequency and flapping amplitude are given, the positive thrust and negative thrust generated in one flapping cycle are significantly different. The conventional nondeformable mode generates the most negative thrust in one cycle, followed by the sinusoidal-based mode. The instant mode has the least negative thrust, and this is why it has the best thrust performance. This work provides a new way to design and control the caudal fin of biomimetic robotic fish in fulfillment of large thrust generation.

In the future, more explorations will be conducted to study the deformation of the caudal fin, such as Computational Fluid Dynamics (CFD), real-time closed-loop control, and pragmatic optimization of the design.

## Figures and Tables

**Table 1:** Three deformation patterns of the caudal fin.

(*A*, *f*, *G*, *B*)	Deformation patterns
(*A*, *f*, 0, −2)	Conventional nondeformable mode
(*A*, *f*, *G*, 0)	Sinusoidal-based mode
(*A*, *f*, 0.25, 25)	Instant mode

**Table 2:** High-level control command in conventional nondeformable mode.

*A*	*f*	*G*	*B*
35°, 40°, 45°, 50°	0.20 Hz	0	−2
35°, 40°, 45°, 50°	0.25 Hz		
35°, 40°, 45°, 50°	0.30 Hz		

**Table 3:** High-level control command in sinusoidal-based mode.

*A*	*f*	*G*	*B*
35°, 40°, 45°, 50°	0.20 Hz	0, 1/8, 2/8, 3/8, 4/8, 5/8, 6/8, 7/8	0
35°, 40°, 45°, 50°	0.25 Hz		
35°, 40°, 45°, 50°	0.30 Hz		

**Table 4:** High-level control command in instant mode.

*A*	*f*	*G*	*B*
35°, 40°, 45°, 50°	0.20 Hz	0.25	25
35°, 40°, 45°, 50°	0.25 Hz		
35°, 40°, 45°, 50°	0.30 Hz		

**Table 5:** Mean thrust comparison of sinusoidal-based mode to conventional nondeformable mode.

*f*	*A*			
	35°	40°	45°	50°
0.20 Hz	8.3%	7.2%	18.2%	14.4%
0.25 Hz	3.0%	2.6%	1.6%	−0.5%
0.30 Hz	0.5%	2.2%	1.0%	5.3%

**Table 6:** Mean thrust comparison of instant mode to conventional nondeformable mode.

*f*	*A*			
	35°	40°	45°	50°
0.20 Hz	19.4%	20.6%	27.5%	22.1%
0.25 Hz	6.4%	15.3%	22.3%	13.8%
0.30 Hz	2.4%	7.2%	5.7%	10.9%

## Data Availability

The datasets generated during and/or analyzed during the current study are available from the corresponding author on reasonable request.
